# Is there a correlation between socioeconomic disparity and functional outcome after acute ischemic stroke?

**DOI:** 10.1371/journal.pone.0181196

**Published:** 2017-07-26

**Authors:** Tian Song, Yuesong Pan, Ruoling Chen, Hao Li, Xingquan Zhao, Liping Liu, Chunxue Wang, Yilong Wang, Yongjun Wang

**Affiliations:** 1 Department of Neurology, Beijing Tiantan Hospital, Capital Medical University, Beijing, China; 2 China National Clinical Research Center for Neurological Diseases, Beijing, China; 3 Center of Stroke, Beijing Institute for Brain Disorders, Beijing, China; 4 Beijing Key Laboratory of Translational Medicine for Cerebrovascular Disease, Beijing, China; 5 Department of Epidemiology and Health Statistics, School of Public Health, Capital Medical University, Beijing, China; 6 Beijing Municipal Key Laboratory of Clinical Epidemiology, Beijing, China; 7 Centre for Health and Social Care Improvement, Faculty of Education Health and Wellbeing, University of Wolverhampton, Wolverhampton, United Kingdom; 8 Post Graduate Academic Institute of Medicine, University of Wolverhampton, Wolverhampton, United Kingdom; Massachusetts General Hospital, UNITED STATES

## Abstract

**Background:**

To investigate the impact of low socioeconomic status (SES), indicated by low level of education, occupation and income, on 3 months functional outcome after ischemic stroke.

**Methods:**

We analyzed data from the China National Stroke Registry (CNSR), a multicenter and prospective registry of consecutive patients with acute cerebrovascular events occurred between September 2007 and August 2008. 11226 patients with ischemic stroke had SES and clinical characteristics data collected at baseline and mRS measured as indicator of functional outcome in 3 months follow up. Multinomial and ordinal logistic regression models were performed to examine associations between SES and the functional outcome.

**Results:**

At 3 months after stroke, 5.3% of total patients had mRS scored at 5, 11.3% at score 4, 11.1% at score 3, 14.4% at score 2, 34.2% at score 1 and 23.7% at score 0. Compared to patients with educational level of ≥ 6 years and non-manual laboring, those < 6 years and manual laboring tended to have higher mRS score (P<0.001). Multinomial adjusted odds ratios (ORs) of outcome in manual workers were significantly increased (ORs from1.38 to 1.87), but OR in patients with less income was not significant. There were similar patterns of association The impact may be stronger in patients aged <65 years (P = 0.003, P<0.001 respectively) and being male (P = 0.001, P<0.001 respectively).

**Conclusions:**

Our study provides evidence that people who are relatively more deprived in socioeconomic status suffer poorer outcome after ischemic stroke. The influence of low educational level and manual laboring can be more intensive than low income level on 3-month outcome. Health policy and service should target the deprived populations to reduce the public health burden in the society.

## Introduction

Stroke is a leading cause of disability and mortality in the world [1,2]. Over the past two decades, the burden of stroke has risen to the third among 291 diseases and injuries [1]. Previous studies have shown that people with low socioeconomic status (SES) have an increased incidence of stroke [3] and a higher mortality after stroke [4–7]. However, it is unclear whether low SES is associated with increased functional impairment after stroke. There is less investigation in this area. Recently a study from the UK has shown that low SES was associated with functional impairment at 3 months after stroke [8]. But the association was not significant in younger and male patients. The impact of SES on functional impairment after stroke needs more research. In this study, we examined data of a large-scale cohort study in China to investigate individual and combined impacts of each of educational level, occupational class and income on functional impairment after stroke.

## Materials and methods

### Data source: The China National Stroke Registry (CNSR)

All data in this paper were obtained from the largest stroke registry in China, CNSR, which was a multicenter and prospective registry of consecutive patients with acute cerebrovascular events from September 2007 to August 2008[9]. The CNSR included up to 132 hospitals covering 27 provinces and 4 municipalities, which is equal to 80% geographical area of the whole China. Well-trained research coordinators at each hospital reviewed medical records daily to identify, consent and enroll consecutive patients. To be eligible for this study, subjects had to meet the following criteria: (1) age 18 years or older; (2) hospitalized with a primary diagnosis of acute ischemic stroke according to the World Health Organization (WHO) criteria[10]; (3) stroke confirmed by head computerized tomography (CT) or brain magnetic resonance imaging (MRI); (4) direct admission to hospital from a physician’s clinic or emergency department; (5) written informed consent from patients or their legal representatives.

The CNSR protocol was approved by a central medical ethics committee at Beijing Tiantan Hospital and research board of each participating center. All patients or their legal representatives provided written informed consent before enrollment.

### Data collection and definition of variables

In the CNSR network, a standardized case report form was used for data collection. The relevant data were accurately recorded. For this study, the following variables were analyzed: demographics (age and gender); modified Rankin Scale (mRS); stroke risk factors: hypertension (history of hypertension or anti-hypertensive medication record), diabetes mellitus (history of diabetes mellitus or anti-diabetic medication record), dyslipidemia (history of dyslipidemia or lipid-lowering medication record), cardiovascular disease, atrial fibrillation (history of atrial fibrillation or documentation of atrial fibrillation on admission), history of stroke and mRS score before this stroke, smoking status and heavy alcohol consumption (≥2 standard alcohol beverages per day); admission stroke severity based on the National Institutes of Health Stroke Scale (NIHSS) score; stroke subtype defined by the Trial of Org 10172 in Acute Stroke Treatment (TOAST) classification[11].

### Definition of SES indicators

We selected education level, occupational class and monthly income as variants reflecting SES [12,13]. Information on SES was collected through the registry. Educational levels were summarized into 3 categories according to years in full-time education: “>9 years (senior high school /college/university degree)”, “6–9 years (junior high school)” and “<6 years (primary school degree)”. Patients’ occupational classes were determined as “non-manual workers”, “manual workers”, “no job” and “retired” according to their last jobs. Monthly income was categorized to 2 grades according to the disposable income per capita: “≤$160” and “>$160”. Based on previous literature [14], the patients with <6 years education, no job or manual workers, and individual income ≤$160 per month were defined as socioeconomic deprivation (SED) in our study.

### Functional outcome assessment at 3 months

A central follow-up team blinded to baseline information conducted telephone interview based on a standardized interview protocol. The mRS at 3 months after stroke was recorded to evaluate functional outcome, which is scaled to 6 points ranging from 0 (no symptoms) to 5 (severe disability).

### Statistical analysis

First, descriptive statistics were used to provide information on the patients’ demographic, socioeconomic, and clinical characteristics. Continuous variables such as age and NIHSS score were presented as mean±SD and median with interquartile range respectively. We used chi-square test to evaluate the statistical difference of SES between mRS groups.

Second, we employed multinomial logistic regression models to examine the association of SES with different levels of disability, defined by each of the mRS score categories. The association was expressed as odds ratios (ORs) with corresponding 95% confidence intervals (CIs). The ORs were adjusted for potential confounding covariates, including age, gender, hospital, risk factors, stroke subtype, NIHSS score on admission, stroke unit admission, swallow test, and medications.

Third, we used ordinal logistic regression to estimate the association of SES indicators with functional outcome in a common OR. The common OR measured the likelihood for a shift in the direction of a poor outcome on the mRS. Common ORs were adjusted for potential confounding covariates, including age, gender, hospital, smoking status, heavy alcohol drinking, cardiovascular diseases and risk factors score, previous stroke, pre-stroke mRS, 5 medications before admission/ in hospital/ on discharge, stroke subtype, NIHSS score on admission, stroke unit admission, swallow test. After examining the association of functional outcome with each single SED indicator, we investigated combined effects of pairwise indicators (education with occupation, education with income, or occupation with income), and then all 3 indicators (scores summed up from occupation, education and income). We compared the impacts of SES on 3-month functional impairment between younger and older patients and between men and women according to literature[8].

In these multivariate logistic regression analyses, missing values for education, occupational class and income level were imputed using multiple imputation techniques. We generated 5 imputed data sets, and the ORs were then averaged across the 5 imputations. Missing values for other covariates were treated as a separate group in the models without imputation.

All tests were 2-sided, and statistical significance was determined at an alpha level of 0.05. All statistical analyses were performed with SAS software version 9.3 (SAS Institute Inc, Cary, NC).

## Results

**Fig 1:**
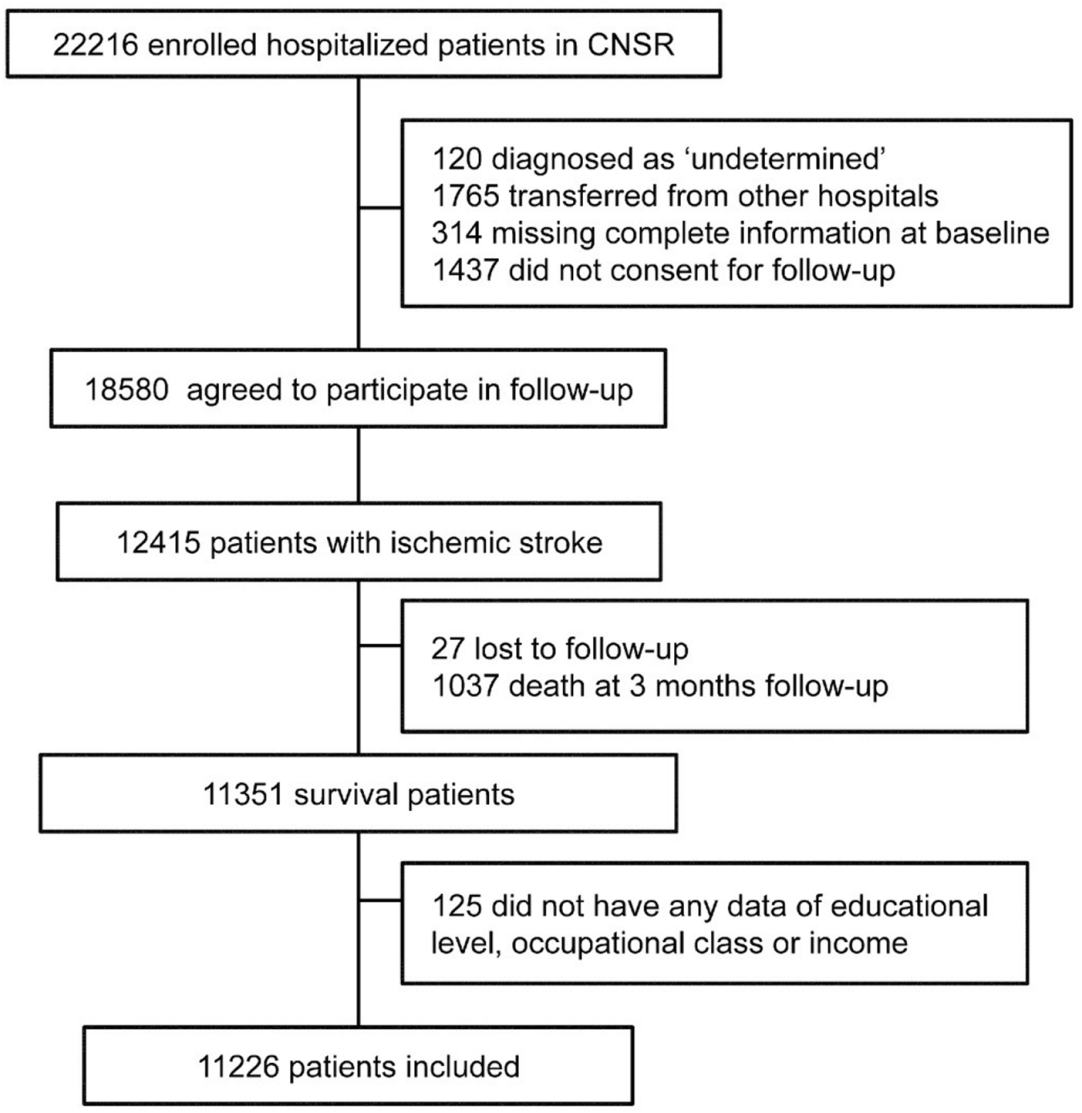
Flow diagram of participants.

A total of 11,226 patients were enrolled in the final statistical analysis (Fig 1), including 2,661 patients with mRS scored as 0, 3,844 as mRS 1, 1,611 as 2, 1,250 as 3, 1,267 as 4 and 593 as 5. Their characteristics were shown in S1 Table. We found that mRS score increased with age, female gender and atherosclerosis risk factors.

Table 1 showed the number and percentage of each of 3 SES indicators among patients with different mRS scores. There was an inverse association between educational level and mRS score; patients with education at <6 years had a significantly higher score of mRS. Patients with high mRS score were likely to be unemployed or retired, and the association of low income with mRS approached a conventional significance in the univariate analysis.

**Table 1:** SES of patient according to mRS score in CNSR, n (%).

Socioeconomic status	mRS 0 (N = 2661)	mRS 1 (N = 3844)	mRS 2 (N = 1611)	mRS 3 (N = 1250)	mRS 4 (N = 1267)	mRS 5 (N = 593)	P*
**Educational level (years)**							
>9	867(32.6)	1215(31.6)	443(27.5)	272(21.8)	305(24.1)	118(19.9)	<0.001
6–9	690(25.9)	981(25.5)	386(24.0)	290(23.2)	255(20.1)	120(20.2)	
<6	845(31.8)	1294(33.7)	639(39.7)	565(45.2)	574(45.3)	304(51.3)	
Unknown	259(9.7)	354(9.2)	143(8.9)	123(9.8)	133(10.5)	51(8.6)	
**Occupational class**							
Non-manual workers	585(22.0)	729(19.0)	215(13.3)	158(12.6)	136(10.7)	47(7.9)	<0.001
Manual workers	655(24.6)	1118(29.1)	483(30.0)	376(30.1)	275(21.7)	119(20.1)	
No job	245(9.2)	321(8.3)	187(11.6)	176(14.1)	158(12.5)	83(14.0)	
Retired	1074(40.4)	1537(40.0)	673(41.8)	500(40.0)	653(51.5)	324(54.6)	
Unknown	102(3.8)	139(3.6)	53(3.3)	40(3.2)	45(3.6)	20(3.4)	
**Income level ($/month)**							
>160	1125(42.3)	1626(42.3)	660(41.0)	487(39.0)	519(41.0)	245(41.3)	0.09
≤160	905(34.0)	1342(34.9)	570(35.4)	475(38.0)	415(32.7)	193(32.6)	
Unknown	631(23.7)	876(22.8)	381(23.6)	288(23.0)	333(26.3)	155(26.1)	

*Overall *P* value for the variable.

Multinomial logistic model analysis (Table 2) showed associations of low SES with mRS. Multivariate adjusted ORs in patients with mRS 2 to 5 verse those with mRS 0 were significantly increased with educational level of <6 years. Patients with manual laboring had an increased odds of disability states of mRS 1 to 5, while those unemployed had increased ORs of mRS 2 to 4. Patients with income ≤160 $/month had a significantly increased OR of mRS 3.

**Table 2:** Adjusted odds ratios of 3-month mRS after stroke.

Socioeconomic status	mRS 1 OR* (95% CI)	P †	mRS 2 OR* (95% CI)	P †	mRS 3 OR* (95% CI)	P †	mRS 4 OR* (95% CI)	P †	mRS 5 OR* (95% CI)	P †
**Educational level (years)**										
>9	1		1		1		1		1	
6–9	1.05(0.92–1.21)	0.46	1.13(0.94–1.36)	0.18	1.33(1.06–1.66)	0.02	1.05(0.83–1.33)	0.66	1.33(0.95–1.87)	0.10
<6	1.07(0.92–1.25)	0.35	1.27(1.06–1.53)	0.01	1.59(1.27–1.98)	<0.001	1.25(1.01–1.55)	0.04	1.45(1.08–1.96)	0.01
**Occupational class**										
Non-manual workers	1		1		1		1		1	
Manual workers	1.38(1.17–1.63)	<0.001	1.87(1.50–2.33)	<0.001	1.77(1.36–2.30)	<0.001	1.51(1.13–2.01)	0.006	1.81(1.16–2.82)	0.01
No job	0.99(0.79–1.24)	0.96	1.70(1.29–2.24)	<0.001	1.81(1.32–2.48)	<0.001	1.42(1.01–1.99)	0.047	1.47(0.90–2.41)	0.13
Retired	1.07(0.91–1.25)	0.42	1.48(1.18–1.84)	<0.001	1.21(0.94–1.56)	0.14	1.25(0.95–1.64)	0.11	1.55(1.02–2.34)	0.04
**Income level ($/month)**										
>160	1		1		1		1		1	
≤160	1.01(0.88–1.15)	0.88	1.06(0.89–1.26)	0.53	1.22(1.02–1.45)	0.03	1.23(0.98–1.54)	0.09	1.27(0.98–1.66)	0.08

*Model 1– MI, multinomial logistic regression adjusted for age, gender, hospital, smoking status, heavy alcohol drinking, cardiovascular diseases and risk factors score [hypertension + diabetes mellitus + dyslipidemia + coronary heart disease + atrial fibrillation], previous stroke, pre-stroke mRS, 5 medications before admission, stroke subtype, NIHSS score on admission, stroke unit admission, swallow test, 5 medications in hospital and 5 medications on hospital discharge.

†Overall *P* value for the variable.

Multivariate adjusted ordinal logistic model analysis (Table 3) showed an overview OR across 6 mRS categories from 0 to 5 scores. There was a statistically significant shift toward poorer outcomes in the distribution of the score of mRS for patients with educational years <6 (OR 1.20, 1.09–1.32), manual workers (OR 1.31, 1.16–1.47), those without job (OR 1.31, 1.13–1.52) and monthly income ≤160 $ (OR 1.14, 1.04–1.24).

**Table 3:** Adjusted common odds ratios of 3-month mRS after stroke.

Socioeconomic status	cOR* (95% CI)	*P* †
**Educational level (years)**		
>9	1	
6–9	1.08(0.97–1.20)	0.18
<6	1.20(1.09–1.32)	<0.001
**Occupational class**		
Non-manual workers	1	
Manual workers	1.31(1.16–1.47)	<0.001
No job	1.31(1.13–1.52)	<0.001
Retired	1.13(1.01–1.27)	0.04
**Income level ($/month)**		
>160	1	
≤160	1.14(1.04–1.24)	0.004

*Model 2– MI,ordinal logistic regression adjusted for age, gender, hospital, smoking status, heavy alcohol drinking, cardiovascular diseases and risk factors score [hypertension + diabetes mellitus + dyslipidemia + coronary heart disease + atrial fibrillation], previous stroke, pre-stroke mRS, 5 medications before admission, stroke subtype, NIHSS score on admission, stroke unit admission, swallow test, 5 medications in hospital and 5 medications on hospital discharge.

†Overall *P* value for the variable.

The data analysis stratified by age and gender showed that the magnitude of the impact of SED on function outcome in younger patients was greater than that in older patients. No significant effect of SED on function recovery was found in women (P value ranging from 0.04–0.61). For men, those non-manual workers had better outcome than their counterparts (P<0.05) (Table 4).

**Table 4:** Adjusted common odds ratios of 3-month mRS after stroke in young & older patients and men & women.

Socioeconomic status	Age<65 OR* (95% CI)	*P* †	Age> = 65 OR* (95% CI)	*P* †	Men OR* (95% CI)	*P* †	Women OR* (95% CI)	*P* †
**Educational level (years)**								
>9	1		1		1		1	
6–9	1.19(1.03–1.36)	0.02	1.04(0.90–1.20)	0.62	1.06(0.94–1.19)	0.37	1.16(0.97–1.40)	0.10
<6	1.29(1.10–1.51)	0.003	1.29(1.13–1.47)	<0.001	1.23(1.09–1.39)	0.001	1.17(0.98–1.39)	0.08
**Occupational class**								
Non-manual workers	1		1		1		1	
Manual workers	1.38(1.20–1.59)	<0.001	1.29(1.06–1.57)	0.01	1.34(1.17–1.53)	<0.001	1.28(1.02–1.62)	0.04
No job	1.54(1.26–1.88)	<0.001	1.25(1.01–1.56)	0.04	1.38(1.12–1.70)	0.003	1.17(0.92–1.49)	0.21
Retired	1.33(1.15–1.55)	<0.001	1.18(1.00–1.40)	0.056	1.25(1.09–1.42)	0.001	0.94(0.76–1.18)	0.61
**Income level ($/month)**								
>160	1		1		1		1	
≤160	1.16(1.02–1.32)	0.03	1.10(0.98–1.23)	0.10	1.13(1.01–1.27)	0.03	1.13(0.99–1.29)	0.07

*Model–Same with Model 1.

†Overall *P* value for the variable.

Table 5 showed the combined effects of educational level, occupational class on the overall OR of 6 mRS categories in all patients. We found that no matter what the educational level was, patients with low occupational class (manual laboring or no job) or low income had an increased OR. It appears that the impacts of low occupational class or low income on increased functional impairment were greater in patients with high education than those having <6 years education. In the analysis for combined data of income and occupation, we found that patients with low income and low occupation had a highest OR (S3 Table).

**Table 5:** Combined effects of educational level with occupational class or income on 3-month mRS.

Socioeconomic Status	Educational Level ≥6 years OR* (95% CI)	*P* †	Educational Level < 6 years OR* (95% CI)	*P* †
**Occupational class**				
Non-manual workers	1		1.28(0.99–1.65)	0.055
Manual workers	1.31(1.14–1.52)	<0.001	1.41(1.22–1.63)	<0.001
No job	1.45(1.14–1.84)	0.004	1.35(1.12–1.63)	0.002
Retired	1.14(1.002–1.29)	0.047	1.28(1.10–1.50)	0.002
**Income level ($/month)**				
>160	1		1.18(1.05–1.33)	0.009
≤160	1.15(1.02–1.29)	0.03	1.26(1.12–1.42)	<0.001

*Model–Same with Model 2.

†Overall *P* value for the variable.

When all three SED indicators were combined for analysis, multivariate adjusted OR was 1.14 (1.02–1.29) in patients having any one of 3 SEDs, 1.29 (1.15–1.46) in any two of 3 SEDs and 1.38 (1.21–1.57) in all 3 SEDs, trend p<0.001(Table 6).

**Table 6:** Number and adjusted OR of 3-month mRS in relation to SED combinations.

Total score of SED combined*	OR^†^ (95% CI)	*P*
**0**	Ref.	
**1**	1.14(1.02–1.29)	0.04
**2**	1.29(1.15–1.46)	<0.001
**3**	1.38(1.21–1.57)	<0.001

* Added scores from each of low levels of educational years <6 (1 score), occupational class (no job or manual workers) (1 score) and income ≤160 $ (1 score).

^†^ The same adjustment with Model 2.

## Discussion

In this large and representative study of stroke register cohort in China, we found that low SES was associated with 3 months functional impairment after stroke. There was a stronger SED impact on poor functional recovery in younger and male patients than in older and female patients. There is limited data in the worldwide literature to examine the association between low SES and functional recovery after stroke. Previous studies showed inconsistent findings of the association. In the Netherlands, van den BosGA et al examined a small sample of stroke patients (n = 465) and found that there was no significant association of low level of education with disability (Barthel Index and mRS) at 6 months and 5 years post-stroke [15]. A study of small sample (n = 175) in Spain reported that socioeconomic level did not affect patients' functional recovery in 6 months after stroke [16]. In Europe, Putman et al analyzed the data of 419 consecutive patients with stroke and got conflicting results: during inpatient stay, functional impairment 6 months after stroke was associated with a low educational level but not low income, and between discharge and 6 months post stroke, no significant association was found with the educational level and income [17]. However, The Berlin Stroke Register data, including 1688 patients with ischemic stroke only, showed that patients with lower education level had lower rate of functional recovery 3 months after stroke, which could not fully be explained by variations in the patients’ clinical and demographic characteristics [18]. In the South London Stroke Register cohort study we also found that SED was associated with short- (3 months) and long-term (3 years) functional impairment after stroke [8] and the effect of SED on poor functional recovery was predominant among patients who were older and women [8]. Our CNSR findings were consistent with those in Germany and the UK. Differences in the impact of SES on functional recovery after stroke in age and gender require further research.

To present the association between SED and functional outcome of ischemic stroke from different perspectives, we performed comprehensive analyses using different regression models with different definitions of outcome variables. While the multinomial logit model was used to explain the impact of SES on different extent of functional impairment, the ordinal logistic regression was used to estimate common OR which measured the likelihood for a shift in the direction of a poor outcome on the mRS. Investigators in previous studies used a cut-off point of mRS (eg, mRS of 3–5) as a poor functional recovery [19] for data analysis in a binary logistic regression model. We also carried out such an analysis (S2 Table) and found that the results were similar to those in the ordinal logistic regression analysis. Our CNSR data has provided robust evidence that there was a significant association of low SES with increased functional impairment after ischemic stroke.

The reason why patients with lower SES had poorer functional outcome remains unclear. Results from some previous studies suggested inequality in post stroke outcome might be attributable to differences in stroke severity. Patients with lower socioeconomic status were reported to suffer from more severe strokes [15,20,21], therefore had poorer functional outcome after stroke onset. By contrast, although we did find a remarkable inequality in stroke severity defined by NIHSS score on admission (S1 Table), lower SES is still significantly associated with stronger functional deficit 3 months after stroke after adjusting variables such as age, gender, vascular risk factors, NIHSS score on admission and other covariates (Tables 2–5). In other words, the association between SES and outcome cannot be explained sufficiently by stroke severity in our investigation.

Another reason could be that SES influences the efficiency of stroke management during inpatient and after discharge. Patients with lower SES have less probability to attain standard drug therapy, high-quality rehabilitation and nursing care [22,23]. A study from Denmark (14 545 people) showed that low SES was associated with reduced odds of having good-quality acute treatment (admission to stroke unit; imaging; physiotherapy and occupational therapy assessment; and antiplatelet treatment) [24]. This difference existed whether socioeconomic status was measured as income, education, or occupation. Therefore, SES maybe serve as an independent factor to impact the functional outcome of stroke.

China is the largest low- and middle-income country in the world and has the largest number of stroke patients. Over the past three decades, China has experienced extraordinary social and economic changes, which is accompanied with an increase in socioeconomic inequality [25,26]. Our findings will contribute to public health policy focusing on the SES disadvantaged populations to improve their stroke outcome.

To our knowledge, CNSR is the first study from the low and middle-income countries to report the association between SES and functional recovery after stroke. It is one of the largest cohorts of patients with ischemic stroke in the world to be used for examining the association between SES and functional impairment. This offered us good opportunities to examine the associations in subgroups of population of stroke. We employed multinominal and ordinal logistic regression models for data analysis, which could examine each of increased mRS in relation to SES.

Our study has limitations. First, the CNSR dataset did not cover the hospitals in rural areas and missed more patients who had lower levels of education, occupation and incomes [27,28]. Most rural hospitals lacked essential equipment and staff to handle acute stroke, such as CT, rtPA and neurologists. Therefore, rural hospitals were not included in CNSR. This would make our estimation of the impact of SES on functional impairment to be more conservative. Second, the lack of face to face follow-up by neurologists might have some influence on the accuracy of the mRS score at 3 months. Some patients might be unable to report their own situations exactly. Third, the CNSR data we used for analysis in this study is 8–9 years old. It is the only available large-scale representative data evaluating the impact of SES on functional recovery after stroke in China. We checked the literature on this topic and found that no recent other studies in China to examine this important topic. Based on the evidence that there has been an increasing gap between poorer and richer in China [29], we believe that the findings of the current study are of importance for making health policy changes [30]. Finally, like other studies [8,30], we did not further adjust for variables of stroke care after discharge and stroke recurrence. The residuals of the association between SES and mortality might exist, but would be minimized because they are related to other baseline risk factors such as smoking and acute care, which were included for adjustment.

## Conclusions

Our CNSR study has provided the evidence that less educational attainment, unemployment/low occupational class and low income are associated with worse functional outcome after ischemic stroke. On the basis of our findings, health policy and service should target the SES disadvantaged populations to reduce health burden and cost of stroke and improve their stroke outcome.

## Supporting information

S1 Table (DOC)

S2 Table (DOC)

S3 Table (DOC)
