# Supplementary material for: Is there a correlation between socioeconomic disparity and functional outcome after acute ischemic stroke?
Source: PLoS One. 2017 Jul 26;12(7):e0181196. doi: 10.1371/journal.pone.0181196 (PMC5528884; doi:10.1371/journal.pone.0181196)
Supplement: S1 Table — (DOC) [file pone.0181196.s001.doc]

**S1 Table. Characteristics of patients and 3-month outcome in CNSR, n (%).**

| **Variable** | **mRS 0 (N=2661)** | | **mRS 1 (N=3844)** | | **mRS 2 (N=1611)** | | **mRS 3 (N=1250)** | | **mRS 4 (N=1267)** | | **mRS 5 (N=593)** | | **p** | |
| --- | --- | --- | --- | --- | --- | --- | --- | --- | --- | --- | --- | --- | --- | --- |
| **Demography** |  | |  | |  | |  | |  | |  | |  | |
| **Age (year), mean(SD)** | 62.6±12.2 | | 63.1±11.9 | | 64.8±12.3 | | 65.9±11.8 | | 70.1±10.8 | | 73.5±10.6 | | <0.001 | |
| **Sex, n (%)** |  | |  | |  | |  | |  | |  | |  | |
| Men | 1741(65.4) | | 2576(67.0) | | 1028(63.8) | | 723(57.8) | | 663(52.3) | | 302(50.9) | | <0.001 | |
| Women | 920(34.6) | | 1268(33.0) | | 583(36.2) | | 527(42.2) | | 604(47.7) | | 291(49.1) | |  | |
| **Cardiovascular disease and risk factors** | |  | |  | |  | |  | |  | |  | |  |
| **Smoking status, n (%)** | |  | |  | |  | |  | |  | |  | |  |
| Never-smoking | | 1457(54.7) | | 2086(54.3) | | 869(53.9) | | 752(60.2) | | 808(63.8) | | 401(67.6) | | <0.001 |
| Former-smoking | | 337(12.7) | | 462(12.0) | | 234(14.5) | | 188(15.0) | | 167(13.2) | | 95(16.0) | |  |
| Current smoking | | 806(30.3) | | 1196(31.1) | | 470(29.2) | | 279(22.3) | | 257(20.3) | | 84(14.2) | |  |
| Unknown | | 61(2.3) | | 100(2.6) | | 38(2.4) | | 31(2.5) | | 35(2.7) | | 13(2.2) | |  |
| **Heavy drink, n (%)** | |  | |  | |  | |  | |  | |  | |  |
| No | | 2273(85.4) | | 3301(85.9) | | 1410(87.5) | | 1136(90.9) | | 1146(90.4) | | 549(92.6) | | <0.001 |
| Yes | | 349(13.1) | | 487(12.6) | | 184(11.4) | | 102(8.2) | | 96(7.6) | | 32(5.4) | |  |
| Unknown | | 39(1.5) | | 56(1.5) | | 17(1.1) | | 12(0.9) | | 25(2.0) | | 12(2.0) | |  |
| **Hypertension, n (%)** | |  | |  | |  | |  | |  | |  | |  |
| No | | 951(35.7) | | 1368(35.6) | | 616(38.2) | | 431(34.5) | | 407(32.1) | | 184(31.0) | | 0.02 |
| Yes | | 1668(62.7) | | 2409(62.7) | | 976(60.6) | | 796(63.7) | | 832(65.7) | | 397(67.0) | |  |
| Unknown | | 42(1.6) | | 67(1.7) | | 19(1.2) | | 23(1.8) | | 28(2.2) | | 12(2.0) | |  |
| **Diabetes mellitus, n (%)** | |  | |  | |  | |  | |  | |  | |  |
| No | | 2132(80.1) | | 2993(77.9) | | 1245(77.3) | | 909(72.7) | | 899(71.0) | | 446(75.2) | | <0.001 |
| Yes | | 471(17.7) | | 782(20.3) | | 341(21.2) | | 318(25.4) | | 327(25.8) | | 134(22.6) | |  |
| Unknown | | 58(2.2) | | 69(1.8) | | 25(1.5) | | 23(1.9) | | 41(3.2) | | 13(2.2) | |  |
| **Dyslipidemia, n (%)** | |  | |  | |  | |  | |  | |  | |  |
| No | | 1857(69.8) | | 2658(69.2) | | 1110(68.9) | | 817(65.4) | | 789(62.3) | | 387(65.3) | | <0.001 |
| Yes | | 316(11.9) | | 458(11.9) | | 183(11.4) | | 137(11.0) | | 144(11.4) | | 62(10.4) | |  |
| Unknown | | 488(18.3) | | 728(18.9) | | 318(19.7) | | 296(23.6) | | 334(26.3) | | 144(24.3) | |  |
| **Coronary heart disease, n (%)** | |  | |  | |  | |  | |  | |  | |  |
| No | | 2364(88.8) | | 3371(87.7) | | 1386(86.0) | | 1064(85.1) | | 1017(80.3) | | 478(80.6) | | <0.001 |
| Yes | | 297(11.2) | | 473(12.3) | | 225(14.0) | | 186(14.9) | | 250(19.7) | | 115(19.4) | |  |
| **Atrial fibrillation, n (%)** | |  | |  | |  | |  | |  | |  | |  |
| No | | 2518(94.6) | | 3574(93.0) | | 1497(92.9) | | 1107(88.6) | | 1079(85.2) | | 459(77.4) | | <0.001 |
| Yes | | 143(5.4) | | 270(7.0) | | 114(7.1) | | 143(11.4) | | 188(14.8) | | 134(22.6) | |  |
| **Stroke case, severity and acute care** | |  | |  | |  | |  | |  | |  | |  |
| **Previous stroke, n (%)** | |  | |  | |  | |  | |  | |  | |  |
| No | | 1958(73.6) | | 2662(69.3) | | 1050(65.2) | | 812(65.0) | | 723(57.1) | | 285(48.1) | | <0.001 |
| Yes | | 703(26.4) | | 1182(30.7) | | 561(34.8) | | 438(35.0) | | 544(42.9) | | 308(51.9) | |  |
| **Pre-stroke mRS >1, n (%)** | |  | |  | |  | |  | |  | |  | |  |
| No | | 2535(95.3) | | 3597(93.6) | | 1449(89.9) | | 1081(86.5) | | 1027(81.1) | | 452(76.2) | | <0.001 |
| Yes | | 94(3.5) | | 196(5.1) | | 141(8.8) | | 151(12.1) | | 222(17.5) | | 137(23.1) | |  |
| Unknown | | 32(1.2) | | 51(1.3) | | 21(1.3) | | 18(1.4) | | 18(1.4) | | 4(0.7) | |  |
| **Subtype of stroke^†^, n(%)** | |  | |  | |  | |  | |  | |  | |  |
| Large-artery atherosclerosis | | 1069(40.2) | | 1710(44.5) | | 749(46.5) | | 652(52.2) | | 689(54.4) | | 267(45.0) | | <0.001 |
| Small-vessel occlusion | | 651(24.5) | | 824(21.4) | | 244(15.1) | | 148(11.8) | | 95(7.5) | | 40(6.8) | |  |
| Cardioembolism | | 89(3.3) | | 131(3.4) | | 75(4.7) | | 83(6.6) | | 125(9.9) | | 89(15.0) | |  |
| Other or undetermined | | 95(3.6) | | 143(3.7) | | 85(5.3) | | 54(4.3) | | 29(2.3) | | 18(3.0) | |  |
| Unknown | | 757(28.4) | | 1036(27.0) | | 458(28.4) | | 313(25.1) | | 329(25.9) | | 179(30.2) | |  |
| **NIHSS score on admission, median(IQR)** | | 2(1-4) | | 3(2-6) | | 5(3-8) | | 7(4-11) | | 10(5-14) | | 13(7-20) | | <0.001 |
| **Stroke unit admission, n (%)** | |  | |  | |  | |  | |  | |  | |  |
| No | | 2063(77.5) | | 3106(80.8) | | 1293(80.3) | | 1011(80.9) | | 1011(79.8) | | 480(80.9) | | 0.03 |
| Yes | | 598(22.5) | | 738(19.2) | | 318(19.7) | | 239(19.1) | | 256(20.2) | | 113(19.1) | |  |
| **Brain imaging exam, n (%)** | |  | |  | |  | |  | |  | |  | |  |
| No | | 48(1.8) | | 80(2.1) | | 39(2.4) | | 21(1.7) | | 24(1.9) | | 12(2.0) | | 0.72 |
| Yes | | 2613(98.2) | | 3764(97.9) | | 1572(97.6) | | 1229(98.3) | | 1243(98.1) | | 581(98.0) | |  |
| **Swallow test, n (%)** | |  | |  | |  | |  | |  | |  | |  |
| No | | 1654(62.2) | | 2254(58.6) | | 946(58.7) | | 708(56.6) | | 747(59.0) | | 347(58.5) | | 0.047 |
| Yes | | 933(35.1) | | 1449(37.7) | | 613(38.0) | | 493(39.9) | | 468(36.9) | | 230(38.8) | |  |
| Unknown | | 74(2.8) | | 141(3.7) | | 52(3.2) | | 49(3.9) | | 52(4.1) | | 16(2.7) | |  |
| **Five medications score^‡^** | |  | |  | |  | |  | |  | |  | |  |
| **Before hospital admission, n (%)** | |  | |  | |  | |  | |  | |  | |  |
| 0 | | 1162(43.7) | | 1687(43.9) | | 747(46.4) | | 499(39.9) | | 470(37.1) | | 218(36.8) | | <0.001 |
| 1 | | 1007(37.8) | | 1398(36.4) | | 529(32.8) | | 452(36.2) | | 471(37.2) | | 206(34.7) | |  |
| 2 | | 401(15.1) | | 590(15.3) | | 252(15.6) | | 221(17.7) | | 253(20.0) | | 133(22.4) | |  |
| 3 | | 78(2.9) | | 129(3.4) | | 69(4.3) | | 61(4.9) | | 58(4.6) | | 31(5.2) | |  |
| 4-5 | | 13(0.5) | | 40(1.0) | | 14(0.9) | | 17(1.3) | | 15(1.1) | | 5(0.9) | |  |
| **In hospital, n (%)** | |  | |  | |  | |  | |  | |  | |  |
| 0 | | 321(12.1) | | 419(10.9) | | 203(12.6) | | 142(11.4) | | 162(12.8) | | 113(19.1) | | <0.001 |
| 1 | | 633(23.8) | | 938(24.4) | | 373(23.1) | | 288(23.1) | | 309(24.4) | | 173(29.2) | |  |
| 2 | | 944(35.5) | | 1366(35.5) | | 547(34.0) | | 434(34.7) | | 411(32.4) | | 180(30.4) | |  |
| 3 | | 621(23.3) | | 898(23.4) | | 389(24.2) | | 301(24.1) | | 296(23.4) | | 99(16.7) | |  |
| 4-5 | | 142(5.3) | | 223(5.8) | | 99(6.1) | | 85(6.7) | | 89(7.0) | | 28(4.6) | |  |
| **On hospital discharge, n(%)** | |  | |  | |  | |  | |  | |  | |  |
| 0 | | 488(18.3) | | 715(18.6) | | 313(19.4) | | 298(23.8) | | 379(29.9) | | 271(45.7) | | <0.001 |
| 1 | | 582(21.9) | | 787(20.5) | | 326(20.2) | | 235(18.8) | | 240(18.9) | | 116(19.6) | |  |
| 2 | | 865(32.5) | | 1210(31.5) | | 518(32.2) | | 369(29.5) | | 342(27.0) | | 115(19.4) | |  |
| 3 | | 587(22.1) | | 868(22.6) | | 358(22.2) | | 269(21.5) | | 230(18.2) | | 66(11.1) | |  |
| 4-5 | | 139(5.2) | | 264(6.8) | | 96(6.0) | | 79(6.4) | | 76(6.0) | | 25(4.2) | |  |

SD, Standard Deviation; IQR, Interquartile Range; mRS, modified Rankin Scale; NIHSS, National Institutes of Health Stroke Scale.

† Stroke subtype was defined by the Trial of Org 10172 in Acute Stroke Treatment (TOAST) classification.

‡ Antihypertensive, Antidiabetic, Lowering-liquid, Antiplatelet and Anticoagulant.
