# Supplementary material for: Is there a correlation between socioeconomic disparity and functional outcome after acute ischemic stroke?
Source: PLoS One. 2017 Jul 26;12(7):e0181196. doi: 10.1371/journal.pone.0181196 (PMC5528884; doi:10.1371/journal.pone.0181196)
Supplement: S2 Table — (DOC) [file pone.0181196.s002.doc]

S2 Table. Number and adjusted OR of 3-month dependence after stroke – binary logistic regression model.

| **Socioeconomic**  **status** | **Nos of dependence/**  **patients (%)** | **Model^†^**  **OR (95% CI)** | ***P* value** |
| --- | --- | --- | --- |
| **Educational level (years)** |  |  |  |
| >9 | 766/3535(21.7) | 1 |  |
| 6-9 | 750/3022(24.8) | 1.14(0.97- 1.33) | 0.12 |
| <6 | 1594/4669(34.1) | 1.29(1.11- 1.49) | <0.001 |
| **Occupational class** |  |  |  |
| Non-manual workers | 355/1939(18.3) | 1 |  |
| Manual workers | 796/3145(25.3) | 1.20(1.00- 1.44) | 0.053 |
| No job | 427/1212(35.2) | 1.38(1.11-1.73) | 0.004 |
| Retired | 1532/4930(31.1) | 1.10(0.92-1.31) | 0.30 |
| **Income level ($/month)** |  |  |  |
| >160 | 1633/5969(27.4) | 1 |  |
| ≤160 | 1477/5257(28.1) | 1.21(1.06-1.37) | 0.005 |

^†^ Model 3–MI, adjusted for age, gender, hospital, smoking status, heavy alcohol drinking, cardiovascular diseases and risk factors score [hypertension + diabetes mellitus + dyslipidemia + coronary heart disease + atrial fibrillation], previous stroke, pre-stroke mRS, 5 medications before admission, stroke subtype, NIHSS on admission, stroke unit admission, swallow test, 5 medications in hospital and 5 medications on hospital discharge.
