# Supplementary material for: Is there a correlation between socioeconomic disparity and functional outcome after acute ischemic stroke?
Source: PLoS One. 2017 Jul 26;12(7):e0181196. doi: 10.1371/journal.pone.0181196 (PMC5528884; doi:10.1371/journal.pone.0181196)
Supplement: S3 Table — (DOC) [file pone.0181196.s003.doc]

**S3 Table. Combined effects of income level with occupational class on 3-month mRS.**

| **Socioeconomic**  **status** | **Income level >160$**  **OR^†^ (95% CI)** | **p**† **value** | **Income level ≤160$**  **OR^†^ (95% CI)** | **p**† **value** |
| --- | --- | --- | --- | --- |
| **Occupational class** |  |  |  |  |
| Non-manual workers | 1 |  | 1.20(0.99- 1.45) | 0.07 |
| Manual workers | 1.30(1.08- 1.57) | 0.01 | 1.47(1.27- 1.70) | <0.001 |
| No job | 1.30(1.05-1.61) | 0.02 | 1.49(1.22-1.81) | <0.001 |
| Retired | 1.19(1.03-1.37) | 0.02 | 1.24(1.04-1.47) | 0.02 |

*Model 2– MI, ordinal logistic regression adjusted for age, gender, hospital, smoking status, heavy alcohol drinking, cardiovascular diseases and risk factors score [hypertension + diabetes mellitus + dyslipidemia + coronary heart disease + atrial fibrillation], previous stroke, pre-stroke mRS, 5 medications before admission, stroke subtype, NIHSS score on admission, stroke unit admission, swallow test, 5 medications in hospital and 5 medications on hospital discharge.

†Overall *P* value for the variable.
